# Possible potential of *Astrodaucus* genus in development of anticancer drugs 

**Published:** 2021

**Authors:** Narges Ghorbani Hesari, Zahra Tofighi, Seyedeh Fatemeh Shirmardi, Seyed Mostafa Hashemi, Abbas Hadjiakhoondi, Saied Goodarzi

**Affiliations:** 1 *Department of Pharmacognosy, Faculty of Pharmacy, Tehran University of Medical Sciences, Tehran, Iran*; 2 *Medicinal Plants Research Center, Faculty of Pharmacy, Tehran University of Medical Sciences, Tehran, Iran*

**Keywords:** Astrodaucus, Apiaceae, Biologic, Phytochemistry, Benzodioxole

## Abstract

**Objective:**

Many pharmaceutical factories have dramatically improved the quality of herbal remedies in cancer treatment. The results of somestudies have shown anticancer effect of *Astrodaucus *genus*.* Therefore, the aim of this article was to review the chemical ingredients and biological effects of *Astrodaucus* genus especially *A. persicus *from the family Apiaceae (Umbelliferae).

**Materials and Methods:**

Online databases ScienceDirect, PubMed, Scopus, and Google Scholar were searched using the keywords *Astrodaucus*, Apiaceae, Biologic, Phytochemistry, and Benzodioxole to retrieve studies published between 1970 and 2020.

**Results:**

The *Astrodaucus* genus has two species, *Astrodaucus persicus* (Boiss.) Drude and *Astrodaucus orientalis* (L.) Drude. In this genus, 5 new biologically active phytochemicals with benzodioxole structure were introduced and their biological effects were assessed.

**Conclusion:**

Since many of the most commonly used anticancer drugs such as etoposide, teniposide, podophyllotoxin and sanguinarine have benzodioxole structureand according to the results of biological tests, it seems that more researchwith these perspectives should be done on this genus.

## Introduction

Plants and animals have played a significant role in human life ad their effective ingredients have been used for many years to boost the quality of human life (Abdolmohammadi et al., 2008). The ethnomedical data approach is used in drug discovery and a specific plant is selectedaccording to its use in folk medicine (Lee, 1999). 

Genistein, daidzein, quercetin and apigenin are famous natural phenolic compounds with anticancer properties (Wang et al., 2002; Chen et al., 2003; Ramos, 2007). Apigenin and quercetin show antiangiogenic effect and they could reduce the growth and invasiveness of tumor (Gupta et al., 2010; Zhang et al., 2000).

The Apiaceae family with 300–450 genus and 3000–3700 species in the world , is one of the largest and best known flowering plant families in the world (Amiri et al., 2016). Although the herbs of this family are aromatic and have distinctive flavors, some of them are toxic and had been used for execution in ancient Athens (Amiri et al., 2016). Iran is one of the major centers of diversity for this family. The Apiaceae family is characterized by 121 genera and 360 species in Iran. Apiaceae is also one of the most influential plant families in the flora of Iran with 122 endemic species (Mozaffarian, 2007; Emami et al., 2010). In this family, there are a variety of ornamental and medicinal plants. Some species in the Apiaceaefamily are trusted sources of phytochemicals (Danciu et al., 2013)*A. persicus, Levisticum officinale, Thapsiagarganica, Physospermum verticillatum, *from this family, have been reported to have proapoptotic and antiproliferative effects on different cancer cell lines (Danciu et al., 2013). Perfumed plants from this family are able to producesecondary metabolites such as phenolics, sesquiterpenes and monoterpenes (Bouchekrit et al., 2016). The essential oils (EOs) have antimicrobial and antioxidant properties. Presence of terpenes and their oxygenated compounds caused the activity of the EOs.(Bouchekrit et al., 2016). The plants of Apiaceae family have various biological activities including vasorelaxant, antibacterial, hepatoprotective, antitumor, and COX inhibitory activities and they are able to induce apoptosis (Pae et al., 2002).

This genushas two species in Iran, *Astrodaucus persicus* (Boiss.) Drude and *Astrodaucus orientalis* (L.) Drude (Bazargani et al., 2006). *A. persicus* is chiefly distributed in Mazandaran, Semnan, Tehran and Golestan provinces in Iran (Bazargani et al., 2006).

 In addition to chemical anticancer compounds, several anticancer compounds that act via various mechanisms of action, have been extracted from plant sources, valuable economic plants such as *Taxusbrevifolia, Curcuma longa, Catharanthus roseus, Cephalotaxus* species, *Betula alba, Erythroxylumprevillei,* and many others (Gupta et al., 2017). More than 60% of common anticancer compounds were prepared form the nature (Cragg et al., 2005). In 1950, a group of alkaloids derived from*vinca *and cytotoxic podophyllotoxins were discovered as the first anticancer compounds from plants (Balunas et al., 2005). Many natural compounds with anticancer effects (taxol, vinblastine, vincristine, etc.) were structurally modified to yield more powerful anti-cancer analogues with fewer adverse effects (Srivastava et al., 2005). The National Cancer Institute (NCI) collected about 35,000 plant samples from 20 countries and screened around 114,000 extracts for anticancer activity (Cragg et al., 2005). 

The imbalance between cell proliferation and cellular death is one of the main causes of cancer (Wong, 2011). Since cell cycleregulation is the basic mechanism that determinescellfate, among chemotherapy agents that alter cell cyclehave been of special interest (Dobashi et al., 2003).Drugs such as etoposide, camptothecin, vincristine, cis-platinum, cyclophosphamide, paclitaxel (Taxol), 5- fluorouracil and doxorubicin cause apoptosis in cancer cells (Abdolmohammadi et al., 2008).

In some studies *Astrodaucu spersicus* was tested foranti-cancerproperties. Abdolmohammadi et al. determined the antiproliferative effects of *A.persicus* extracts in comparison to doxorubicin on T47D cells by yellow tetrazolium salt (3-(4,5-dimethylthiazol-2-yl)-2,5-diphenyltetrazolium bromide or MTT method (Abdolmohammadi et al., 2008). The purpose of this paper was to investigate whether it is possible to find anti-cancer molecules from *Astrodaucus* genus based on the available findings.

## Materials and Methods

Online databases Science Direct, PubMed, Scopus, and Google Scholar were searched using the keywords *Astrodaucus, *Apiaceae, Biologic, Phytochemistry*, *and Benzodioxole for articles published between 1970 and 2020.

## Results

Benzodioxoles are important compounds in medicinal chemistry and many drugs with this structural skeleton and different therapeutic effects have been marketed (Wang et al., 2013; Chen et al., 2013). In addition, more biological effects such as anticancer, antibacterial, anti-inflammatory, antioxidant, immune modulatory and antihypertensive effects ofthis group of compounds have been observed (Dawood et al., 2019).

**Essential oils**

Several studies have described the chemical composition of essential oils of species from various origins as follows.

In a study, aerial parts of *A. persicus *were studied. The major components of the aerial parts EO were decanal (34.8%), dodecanal (15.5%) and dodecanol (14.3%), with lesser amount of decanol (9.3%) and carvacrol (8.6%) (Bigdeli et al., 2004).

In another study, the chemical constituents from the root, leaf and aerial part of *A. persicus* were investigated (Bazargani et al., 2006).

Other compounds present in appreciable amounts were α-pinene, β-pinene, thymol methyl ether, carvacrol methyl ether, germacrene D and β-bisabolene in the EO of root, limonene in the stem/leaves EO, β -myrcene and fenchyl acetate in the flowers/fruits EO (Bazargani et al., 2006).

In another study as shown in Table 1, leaves/stems and flowers/fruits were gathered in June and ripe fruits and roots were prepared in September 2010 from Kordestan Province (Goodarzi et al., 2016a). The aerial parts EO samples yielded 0.6-0.9% (v/w) and observed as blue color liquid, while the roots EO was seen as yellow color liquid in yield of 0.1% (v/w) (Goodarzi et al., 2016a).

As can be seen in Table, the amount of α-thujene and α-pinene decreased with maturation in ripe fruits while β-pinene content was increased. 

Three compounds including α-pinene, γ-terpinene and bornyl acetate were typical in aerial parts and roots essential oils. 

α-fenchyl acetate, α-thujene, α-pinene, α-eudesmol, β-eudesmol, p-cymene, γ-terpinene, bornyl acetate, γ-cadinene, and camphene were the major components of three aerial parts EOs.

Sesquiterpenoids in blue aerial parts EOs are β-eudesmol and α-Eudesmol, they did not exist in roots EO color or dehydrogenation of β-eudesmol and α-eudesmol are responsible for blue color.The creation of blue color in ripe fruits EO can be due to the presence of camazulene (0.2%) (Goodarzi et al., 2016a).

The extract of leaves, flowers and stems of another species,* A. orientalis* L. obtained by hydrodistillation, showed that β-pinene (20.5%), α-thujene (8.7%0) and α-pinene (7.6%) were the main constituents of the flowers, sabinene (11.8%), α-pinene (8.7%), and *p*-mycrene (2.5%) for the stem, and α-pinene (9.4%), sabinene (13.5%), β-pinene (6.3%), and *p*-mycrene (3.2%) for the leaf (Torabbeigi et al., 2013). The EOs of another species (*A. orientalis*) leaves and seeds were analyzed by Mirza et al. and the chief components of the leaf EO were fenchylacetate (44.5%) and α-pinene (21.6%), whilethe major constituents ofthe seed EO were myrcene (47.7%) and β-pinene (21.8%). The seed EO was found to contain lower amounts of bornyl acetate, germacrene D and δ-cadinene than the leaf oil (Mirza et al., 2003).

**Table 1:** Color, total componentsand major constituents, percent of various types of terpenes

	**Root**	**Stem/Leaves**	**Fruit/Flower**
Color	Yellow	Green	Bluish Green
Total Components	22	20	14
Major Constituents	Bornylacetate (26.5%) β-sesquiphellandrene (25.9%) *exo*-fenchyl acetate(25.1%)	α-pinene (56.4%) *exo*-fenchylacetate (37.7%)	β-pinene (46.1%) α-pinene (26.1%) α-thujene(14.4%)
Monoterpenes	63.7%	98.8%	99.7%
Sesquiterpenes	30.7%	0.9%	0.2%

In summer 2009, the flowers of *A. orientalis *were collected from Markazi province, Iran. Itconsisted of 15 monoterpene hydrocarbons (61.3%), 19 oxygentatedmonoterpenes (18.3%), 15 sesquiterpene hydrocarbons (4.6%), 9oxygentatedsesquiterpenes (6.3%) and 5nonterpenoids compounds (2.4%). Sabinene (16.5%) and α-pinene (11.0%) were the major components in the flower oil of *Astrodaucus orientalis*, followed by myrcene (7.0%), *p*-cymene (6.1%), α-thujene (6.1%) and β-pinene (5.2%) (Masoudi et al., 2012). Table 3presents the comparative list of major compounds of different parts of *A. orientalis *identified ina study in 2009 (Nazemiyeh et al., 2009). 

It can be seen that geographical origin affects the chemical Constituents of EO. In 2011, the effect of different isolation methods on the quantity and quality of EOs of flowers, stems and leaves of *A.orientalis* was investigated. Methods used in this study included hydrodistillation method (HD), head-space solid-phase microextraction (HS-SPME), and microwave assisted head-space solid-phase microextraction (MA-HS-SPME) (Torabbeigi et al., 2013). Hydrodistillation method was used in previous studies on *A. orientalis* essential oil ( Mazloomifar et al., 2003).

The distribution profile of the constituents of theEO of the stems, the fruits and the umbels of *A. oriantalis *was quite similar, especially considering theoccurrence and quantity of sabinene, myrecene, *para*-cymene, α-pinene, β-pinene, terpineol-4, fenchyl acetate and germacerene D. But, there were considerablevariations in the chemical profiles of the EO of the roots and aerial parts, Phenolic compound slike acetophenone and anisole were found in EO of the roots while they were not present in the EO of the aerial parts (Nazemiyeh et al., 2009). On the basis of findingsfrom previous studies, it is reasonable to state that fenchyl acetate and α-pinene could be used as chemotaxonomic markers inthe species of the genus *Astrodaucus*, at least in two Iranian species (Nazemiyeh et al., 2009). Coumarines were also identified in a solvent extract ofthe aerial parts of *A. orientalis *(Torabbeigi et al., 2013). Determination of the contents of *A. orientalis* showed high amounts of copper (0.47 mg/100 g), manganese (0.90 mg/100 g) and iron (7.12 mg/100 g) (Goodarzi et al., 2016a).

**Biological effects**

**Anti-cancer effects**

When uncontrolled cell proliferation occurs due to the absence of apoptotic signals, it can lead to different types of cancer. About 1.7 million new cancer cases and more than 600,000 deaths were reported in the United States in 2018 (Torre et al., 2018; Bauer et al., 2006). Based on a meta-analysis of 21 retrospective studies, despite chemotherapy, radiation therapy, endocrine therapy, and lumpectomy, the recurrence rate of breast cancer is still high (Houssami et al., 2010).

**Table 2:** Composition of essential oils from different parts of *A.persicus*

	**Root**	**Stem/Leaves**	** Fruit/Flower**	**Ripe Fruit**
Color	Yellow	Blue	---	----
Total Components	21	15	21	24
Major Constituents	Trans-caryophyllene (33.5%) bicycogermacrene (27.3%) germacrene-D (11.6%)	α-thujene (48.0%) α-pinene (27.7%) α-fenchene (9.2%)	α-thujene (43.8%) β-pinene (21.3%) α-pinen (20.9%)	β-pinene (56.9%) α-thujene (17.6%) α-pinene (14.3%)
Monoterpenes	5.2%	96.5%	97.3%	95.5%
Sesquiterpenes	90.7%	2.1%	1.4%	1.1%

**Table 3:** Major compounds and monoterpene hydrocarbons (%) of different parts of *A. orientalis*

	**S** **tem**	**Flower**	**Fruit**	**Root**
Major components	sabinene (23.1%) α-pinene (16.34%) fenchylacetate (7.5%)	α-copaene (26.1%) α-pinene (15.3%) sabinene (13.7%)	sabinene (25.6%) α-pinene (22.3%) α-copaene (16.1%)	Anisole (37.0%) bornyl acetate (36.9%) geranyltiglate (11.4%)
Monoterpene hydrocarbons(%)	(62.7%)	(37.5%)	(57.6%)	_

The apoptotic signals are generated through the intrinsic and the extrinsic pathway. Inhibition of antiapoptotic Pr Bcl-2 and Bcl-Xl expression by stimulating the mitochondrial membrane play major roles inthe intrinsic pathway (Tuorkey, 2014).

An ideal anticancer drug causes death or disability of the cancer cell while not harming normal cells (Taraphdar, 2001). Since the disruption ofthe cell cycle plays an important role in cancer progression, its modulation is attracting great attention. A number of herbs with the ability to induce cell cycle arrest can be effective in preventing and treating cancer. Growing of breast cancer involves activation and deactivation of several types of genes (Ingvarsson, 2001). Wild type p53 is an important regulatory protein in induction of apoptosis after DNA damage induced by anti-cancer drugs. The Bcl-2 is a gene that halts initiation steps of apoptosis and programmed cellular death (Gasco et al., 2003; Krajewski et al., 1999).

In a study, the anticancer effects of *A. persicus*, in human breast cancer T47D cells, were investigated. Also, expression ofp53 and Bcl-2 that are believed to play a critical role in tumorigenesis and cell death, were determined.Results of this study shows that Bcl-2 expression issignificantly increased in the presence of aerial but significantly decreased in the presence of root extractand p53 gene expression significantly increased in the presence of both plant extracts. In addition, treatment of T47D cells with *A. persicus* extracts decreased the nuclear staining of p53 and cytoplasmic staining of Bcl-2 proteins. These results suggest that methanolic fractions especially those from the root, may contain active compounds, probably coumarins that prevent proliferation of T47D breast carcinoma cells by mechanisms such as apoptosis (Azizi et al., 2015). Toxicity of the plant extract and the altered cell cycle pattern were studied (Abdolmohammadi et al., 2008), and the IC50 values of aerial and root extracts on T47D cells were determined and it was shown that both extracts were cytotoxic (1 mg/ml for aerial extract and 0.5 mg/ml for root extract (Abdolmohammadi et al., 2008). Anti-cancer effects of *A. persicus* in human breast cancer T47D cells in comparison to tamoxifen, were evaluated (Azizi et al., 2015). It was found that its efficiencyin cell cycle arrest was not similar to doxorubicin but similar to RPMI control (Abdolmohammadi et al., 2008).

Thus, *in vitro* screening of the extracts (root and aerial parts) showed a time- and dose-dependent inhibition of the cell growth on breast carcinoma T47D cell line (Abdolmohammadi et al., 2008; Tan et al., 2005). Although root extract shows higher anticancer activity in comparison to the extract of aerial part (Abdolmohammadi et al., 2008). But, aerial parts extract of*A. orientalis*, contrary to *A. persicus*, , had higher effects on inducing apoptosis on T47D cell line compared to the root extract (Abdolmohammadi et al., 2009).

In 2015, Goodarzi et al. succeeded in isolation, purification and identification of five pure compounds from different fractions of *A. persicus* root which all had new benzodioxole structures, and two of them contained epoxy unit in their chain structure (Goodarzi et al., 2016b). Benzodioxoles were used as antioxidant, antitumor, antifungal, antibacterial, pesticides, herbicides, antiparasitic and antimalarial agents (Gupta et al., 2016). A number of anticancer drugs with benzodioxole structures showed good bioavailability and low cytotoxicity (Wang et al., 2013). There are some reports on benzodioxole presence in plants. Camphor wood, nutmeg, star anise, mace, parsley and cinnamon leaf (safrole), mace essential oil and other spices of Apiaceae like parsley and dill (Myristicin), celery, parsley and Carumpetroselinum (apiol), dill seed and fennel root (diapiole) are some examples (Buchanan, 1978; Hsuuw et al., 2015).

Subsequent research showed that some of the safrole derivatives were unable to inhibit cell growth, and the antiproliferative effects of these compounds were not only due to the presence of the benzodiaxol ring (Moreira et al., 2007). Epoxy group in the chain is another part of the molecule which increases cytotoxicity in benzodioxole structures. For instance a metabolite of safrole (safrole 2, 3-oxide), induced more potent genotoxic and cytotoxic effects than safrole (Moreira et al., 2007; Chiang et al., 2011).

**Other biological effects**

Essential oils are sources of antimicrobial ingredients, especially against bacterial pathogens. However, antimicrobial activity can be enhanced by a chemical, but in the EOs, this effect appears to be due to synergy among many chemical compounds (Torabbeigi et al., 2012; Prabuseenivasan et al., 2006).

 In 2011, the effects of different isolation approaches on the quality and quantity of EOs of different sections of *A.orientalis *were studied and the antibacterial activities against *Bacillus subtilis* and *Escherichia coli* were investigated. The results of this study showed that the EOs obtained by different extraction methods differed in composition. MICs of the EO of *A. orientalis* L. were determined by the agar dilution method with respect to different test microorganisms, including Gram-negative (*Escherichia coli* PTCC 1330) and Grampositive (*Bacillus subtilis* ATCC 6633) bacteria. These EOs showed good activities against both bacteria (0.5–1.5 mg/ml) (Torabbeigi et al., 2012).

One of the most significant health problems in Iran is malaria, especially in the southern parts of the country (Naddaf et al., 2003). Mosquitoes have a major role in transmission of the disease (James, 1993). *Anopheles stephensi *that is an eastern malaria vector is distributed in countries around the Persian Gulf (Nagpal et al., 1995).

The use of chemical pesticides can lead to occurrence of resistant strains and can pose environmental hazards, accumulation in the food chain, high and acute toxicity, prolonged degradation, and increased potency to eliminate beneficial and harmful pests (Barnard et al., 1997).

Regarding mosquito control methods, several important considerations should be noted: environmental effect, resistance, and cost. Herbal insecticides can be an alternative to chemicals. Most herbal ones are fast acting and break down quickly in the environment. Extracts and EO of some certain plants have been investigated against some public health pests (Hadjiakhoondi et al., 2005; Vatandoost et al., 2008). Some secondary metabolites of plants act as herbal insecticides (Nathan, 2007). Application of natural EO for vector control is a method that reduces the adverse effects of chemical pesticides on the environment (Fatope et al., 1993).

Studies showed that plants from Apiaceae family which contain coumarin compounds can have larvicidal activity. Fruits and roots extracts of *A. persicus* had insecticide potentials (Goodarzi et al., 2017).

In a study by Goodarzi et al., the methanolic extract of the roots was fractionated using hexane (HE), chloroform (CL), ethyl acetate (EA) and methanol (ME) respectively. To determine antioxidant activity of aerial parts EOs and various fractions of root extract, the DPPH and FRAP methods were used. Total root extract and EA fraction showed moderate free-radical scavenging activity. The antioxidant activity of root HE faction and all of aerial parts EO samples were poor as assessed by DPPH method (Goodarzi et al., 2017).

Total antioxidant activity of root fractions and aerial parts EOs was measured according to standard curve of FeSO_4_. Total root extract had the greatest reducing capacity (881.5 mmol Fe^2+^/100 g), which was more than vitamin E (313.7 mmol Fe^2+^/100 g), and comparable with BHA (880.3 mol Fe ^2+^/100 g). The flowers/fruits EO had potent reducing capacity (686.6 mmol Fe^2+^/100 g) higher than vitamin E. The lowest antioxidant activity was observed for HE and methanol (ME) fractions (Goodarzi et al., 2016a).

Total root extract had a potent antioxidant activity in comparison to its fractions. Compared to other species, *A.persicus* root extract showed potent radical scavenging antioxidant activity (Goodarzi et al., 2016a).

Based on the gallic acid standard curve total phenol content of samples wascalculated. Among all samples, total root extract and EA showed the highest content of phenolic compounds. Compared to other species of Apiaceae family such as *Centellaasiatica, Hydrocotylebonariensis*, *H. sibthorpioides* (Abas et al., 2014) and *Cuminumcyminum* L. (Rebey et al., 2012). *A. persicus* demonstrated moderate content of total phenols (Goodarzi et al., 2016a).

There were close positive correlations between the total phenols and FRAP antioxidant activity in root fractions while significant correlations between the amount of total phenols and DPPH antioxidant activity, were not observed (Goodarzi et al., 2016a).

**Figure 1:**
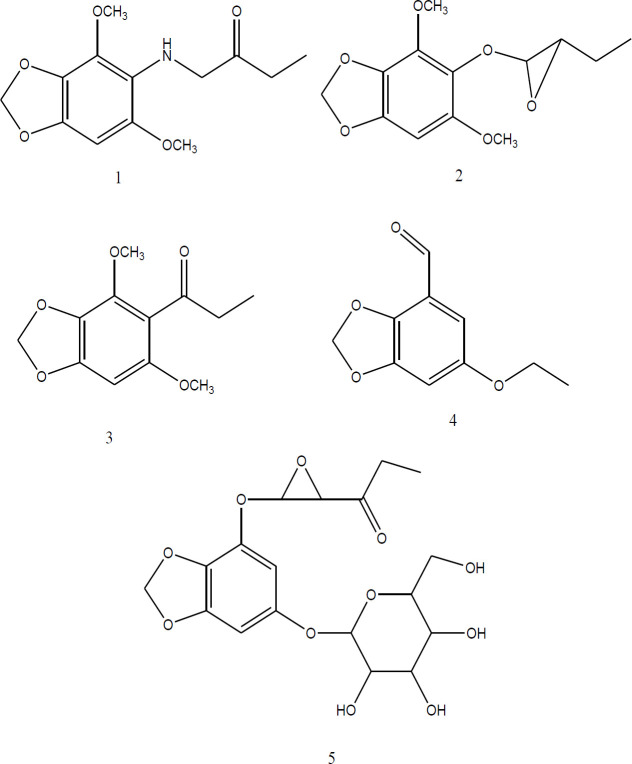
Newly identified compounds with a benzodioxolestructure from *Astrodaucus persicus *(Boiss) Drude

1. 1-(4, 6-dimethoxybenzo[d][1,3]dioxol-5-ylamino)butan-2-one(1)
2. 5-(3-ethyloxiran-2-yloxy)-4,6-dimethoxybenzo[d][1,3]dioxole (2)
3. 1-(4, 6-dimethoxybenzo[d][1,3]dioxol-5-yl)propan-1-one(3)
4. 6-ethoxybenzo[d][1,3]dioxol-4-carbaldehyde(4)
5. 1-(3-(5-hydroxybenzo[d][1,3]dioxol-7-yloxy)oxiran-2-yl)propan-1-one(5)

## Discussion

Cancer is the second leading cause of death around the world, and is responsible for about 1 in 6 deaths. Approximately 70% of deaths from cancer occur in low- and middle-income countries. Therefore, considerable global efforts were made for cancer management.

The need for alternative and less toxic therapies for different kind of cancers, is clear. Based on studies conducted, as a natural remedy,* A. persicus* prevents ontogenesis of T47D breast carcinoma cells by mechanisms such as apoptosis. It seems that* A.persicus *contains compounds that may have anti-cancer effects, probably due to newly identified1, 3-benzodioxole compounds present in this plant. Also, antibacterial, antioxidant and insecticide activitieswere reported.

In terms of chemical composition of essential oils, different isolation methods can affect the number of constituents obtained from the essential oil, and parameters such as geographical origin, climatic conditions and the development stage of the plant affect the chemical composition of volatile oils. 

## Conflicts of interest

The authors have declared that there is no conflict of interest.

## References

[1] Abas F, Khatib A, Shaari K, Shaari K, Lajisae NH, Maulidiani (2014). Chemical characterization and antioxidant activity of three medicinal Apiaceae species. Ind Crops Prod.

[2] Abdolmohammadi MH, Fouladdel SH, Shafiee A, Amin Gh, Ghaffari SM, Azizi E (2008). Anticancer effects and cell cycle analysis on human breast cancer T47D cells treated with extracts of Astrodaucus persicus (Boiss) Drude in comparison to doxorubicin. DARU.

[3] Abdolmohammadi M, Fouladdel S, Shafiee A, AminGh, Ghaffari SM, Azizi E (2009). Antiproliferative and apoptotic effect of Astrodaucus orientalis (L) drude on T47D human breast cancer cell line: Potential mechanisms of action. Afr J Biotechnol.

[4] Amiri M, Joharchi M (2016). Ethnobotanical knowledge of Apiaceae family in Iran: A review. Avicenna J.

[5] Azizi E, Abdolmohammadi MH, Fouladdel SH, Shafiee A, Amin GH, Ghaffari SM (2015). Evaluation of p53 and Bcl-2 genes and proteins expression in human breast cancer T47D cells treated with extracts of Astrodaucus persicus (Boiss ) Drude in comparison to Tamoxifen. DARU.

[6] BalunasMJ, Kinghorn AD (2005). Drug discovery from medicinal plants. Life Sci.

[7] Barnard C, Padgitt M, Uri N (1997). Pesticide use and its measurement. Int. Pest Control.

[8] Bauer JH, Helfand SL (2006). New tricks of an old molecule: lifespan regulation by p53. Aging cell.

[9] Bazargani Y, Almasirad A, Amin G, Shafiee A (2006). Chemical composition of the essential oils of Astrodaucus persicus (Boiss) Drude root, stem/leaves and flowers/fruits. Flavour Fragr J.

[10] Bigdeli M, Abdolhossein R, Ameri N, Masoudi S (2004). Essential Oil of Astrodaucus persicus (Boiss) Drude from Iran. J Essent Oil Res.

[11] Bouchekrit M, Laouer H, Hajji M, Nasri M, Haroutounian SA, Akkal S (2016). Essential oils from Elaeoselinum asclepium: Chemical composition, antimicrobial and antioxidant properties. Asian Pac. J. Trop. Biomed.

[12] Buchanan RL (1978). Toxicity of spices containing methylenedioxybenzene derivatives: a review. J Food Saf.

[13] Chen WF, Huang MH, Tzang CH, Yang M, Wong MS (2003). Inhibitory actions of genistein in human breast cancer (MCF-7) cells. BBA-Mol Basis Dis.

[14] Chen YF, Lin YC, Huang PK, Chan HC, Kuo SC (2013). Design and synthesis of 6,7-methylenedioxy-4-substituted phenylquinolin-2(1H)-one derivatives as novel anticancer agents that induce apoptosis with cell cycle arrest at G2/M phase. Bioorg Med Chem.

[15] Chiang SY, Lee PY, Lai MT, Shen LC, Chung WS, Huang HF, Wu KY, Wu HC (2011). Safrole-2′, 3′-oxide induces cytotoxic and genotoxic effects in HepG2 cells and in mice. Mutat Res Genet Toxicol Environ Mutagen.

[16] Cragg GM, Newman DJ (2005). Plants as a source of anti-cancer agents. J Ethnopharmacol.

[17] Danciu C, Avram S, Gaje P, Pop G, Şoica C, Craina M, Dumitru C, Dehelean C, Peev C (2013). An evaluation of three nutraceutical species in the Apiaceae family from the Western part of Romania: antiproliferative and antiangiogenic potential. J Agroalimentary Processes Technol.

[18] Dawood R, Solaiman A (2019). Synthesis and characterization of new 1,3-benzodioxole derivatives based on Suzuki-Miyaura coupling reaction. Res J Chem Environ.

[19] Dobashi Y, Takehana T, Ooi A (2003). Perspectives on Cancer Therapy: Cell Cycle Blockers and Perturbators. Curr Med Chem.

[20] Fatope M, Ibrahim H, Takeda Y (1993). Screening of higher plants reputed as pesticides using the brine shrimp lethality assay. IJPR.

[21] Emami S, Aghazari F (2010). Iranian endemic phanerogams. Iran J Pharm Res.

[22] Gasco M, Crook T (2003). p53 family members and chemoresistance in cancer: what we know and what we need to know. Drug Resist Updat.

[23] Goodarzi S, Hadjiakhoondi A, Yassa N, Khanavi M, Tofighi T (2016a). Essential oils chemical composition, antioxidant activities and total phenols of Astrodaucus persicus. IJBMS.

[24] Goodarzi S, Hadjiakhoondi A, Yassa N, Khanavi M, Tofighi T (2016b). New benzodioxole compounds from the root extract of Astrodaucus persicus. Iranian journal of pharmaceutical research: IJPR.

[25] Goodarzi S, Vatandoost H, Abai MR, Tavakoli S, Hatamian A, Ajani Y, Hadjiakhoondi A, Yassa N, Tofighi T (2017). Astrodaucus persicus as a new source of bioinsectisides against malaria vector, Anopheles stephensi. Asian Pac J Trop Med.

[26] Gupta A, Khan S, Muzafar M, Yadav AK, Sharma G, Anand R (2017). Anticancer curcumin: natural analogues and structure-activity relationship, in Studies in natural products chemistry.

[27] Gupta SC1, Kim JH, Prasad S, Aggarwal BB (2010). Regulation of survival, proliferation, invasion, angiogenesis, and metastasis of tumor cells through modulation of inflammatory pathways by nutraceuticals. Cancer Metastasis Rev.

[28] Gupta SD, Rao GB, Bommaka MK, Bommaka MK, Raghavendra NM, Aleti S (2016). Eco-sustainable synthesis and biological evaluation of 2-phenyl 1, 3-benzodioxole derivatives as anticancer, DNA binding and antibacterial agents. Arab J Chem.

[29] Hadjiakhoondi A, Vatandoost H, Khanavi M (Abaee MR, Karami M). 2005. Biochemical investigation of different extracts and larvicidal activity of Tagetes minuta L. on Anopheles stephensi larvae. Iran J Pharm Sci.

[30] HoussamiN, Macaskill P, Marinovich ML, Dixon JM, Irwig L, Brennan ME, Solin LJ (2010). Meta-analysis of the impact of surgical margins on local recurrence in women with early-stage invasive breast cancer treated with breast-conserving therapy. Eur J Cancer.

[31] Hsuuw YD, Chan WH (2015). Apoptotic effects of dillapiole on maturation of mouse oocytes, fertilization and fetal development. Drug Chem Toxicol.

[32] IngvarssonS (2001). Breast cancer: introduction. in seminars in CANCER BIOLOGY.

[33] James AA (1992). Mosquito molecular genetics: the hands that feed bite back. Science.

[34] Krajewski S, Krajewski M, Turner BC, Pratt C, Howard B, Zapata JM, Frenkel V, Robertson S, Ionov Y, Yamamoto H, Perucho M, Takayama S, Reed JC (1999). Prognostic significance of apoptosis regulators in breast cancer. Endocr Relat Cancer.

[35] Lee KH (1999). Novel antitumor agents from higher plants. Med Res Rev.

[36] Masoudi S, Fathollahi R, Taherkhani M, Valadkhani Z, Baradari T, Cheraghi M, Rustaiyan A (2012). Volatile Constituents of the Aerial parts of Torilis leptophylla (L) Reichenb Thecocarpus meifolious Boiss Leaves of Xanthogalum purpurascens Ave Lall and Flowers of Astrodaucus orieintalis (L) Drude. Four Umbelliferae Herbs from Iran. J Essent Oil-Bear Plants.

[37] Mazloomifar H, Bigdeli M, Saber-Tehrani M, Rustaiyan A, Masoudi S (2003). Essential Oil of Astrodaucus orientalis (L) Drude. J Essent Oil Res.

[38] Mirza M, Baher Nik Z, Dini M (2003). Chemical composition of the essential oils of Astrodaucus orientalis (L) Drude leaves and seeds. Flavour and fragrance journal.

[39] Moreira DR, Lima Leite AC, Pinheiro Ferreira PM (2007). Synthesis and antitumour evaluation of peptidyl-like derivatives containing the 1, 3-benzodioxole system. Eur J Med Chem.

[40] Naddaf SR, Oshaghi MA, Vatandoost H, Assmar M (2003). Molecular characterization of Anopheles fluviatilis species complex in the Islamic Republic of Iran. EMHJ.

[41] Nagpal B, Sharma V (1995). Indian Anophelines. 66 Janpath, New Delhi 110001.

[42] NathanSS (2007). The use of Eucalyptus tereticornis Sm (Myrtaceae) oil (leaf extract) as a natural larvicidal agent against the malaria vector Anopheles stephensi Liston (Diptera: Culicidae). Bioresour Technol.

[43] Nazemiyeh H, Razavi SM, DelazarA, Asnaashari S, Seyedkhoei N, Daniali S, Nahar L, Sarker S Distribution Profile of Volatile Constituents in Different Parts of Astrodaucus orientalis (L ) Drude. Drude. Rec. Nat Prod.

[44] PaeHO, Hyuncheol Oh, Yun YG, Oh GS, Jang SLL, Hwang KS, Kwon O, Lee HS, Chung HT (2002). Imperatorin, a furanocoumarin from Angelica dahurica (Umbelliferae), induces cytochrome c‐dependent apoptosis in human promyelocytic leukaemia, HL‐60 cells. Pharmacol Toxicol.

[45] PrabuseenivasanS, Jayakumar M, Ignacimuthu S (2006). In vitro antibacterial activity of some plant essential oils. BMC Complement Altern Med.

[46] Ramos S (2007). Effects of dietary flavonoids on apoptotic pathways related to cancer chemoprevention. J Nutr Biochem.

[47] Rebey IB, Zakhama N, Karoui IJ, Marzouk B (2012). Polyphenol composition and antioxidant activity of cumin (Cuminum cyminum L) seed extract under drought. J Food Sci.

[48] Srivastava V, Neg AS, Kumar JK, Gupta MM, Khanuja PS (2005). Plant-based anticancer molecules: a chemical and biological profile of some important leads. Bioorg Med Chem.

[49] Tan ML, Sulaiman SF, Najimuddin N, Samian MR, Tengku Muhammad TS (2005). Methanolic extract of Pereskia bleo (Kunth) DC(Cactaceae) induces apoptosis in breast carcinomaT47-D cell line. J Ethnopharmacol.

[50] Taraphdar AK, Roy M, Bhattacharya R (2001). Natural products as inducers of apoptosis: Implication for cancer therapy and prevention. Current science.

[51] Torabbeigi M, Azar PA, Sharifan A, AghaeiMeibodi Z (2012). Antibacterial activity and comparison of the volatile constituents obtained by several extraction methods from the flowers, stems and leaves of Astrodaucus orientalis. Nat Prod Commun.

[52] Torre LA, Trabert B, DeSantis CE, Kimberly D, Carolyn D, Ahmedin J, Rebecca L (2018). Ovarian cancer statistics. CA Cancer J Clin.

[53] Tuorkey J (2014). Curcumin a potent cancer preventive agent: Mechanisms of cancer cell killing. Interv Med Appl Sci.

[54] Vatandoost H, Khazani A, Rafinejad J, Khoobdel M, Kebriai-Zadeh A, Abai MR, hanafi-bojd AA, Akhavan AA, Abtahi SM, Rafi F (2008). Comparative efficacy of neem and dimethyl phthalate (DMP) against malaria vector, Anopheles stephensi (Diptera: Culicidae). Asian Pac J Trop Med.

[55] Wang HH, Qiu KM, Cui HE, Yang YS, Xing M, Qiu XY, Bai LF, Zhu HL (2013). Synthesis, molecular docking and evaluation of thiazolyl-pyrazoline derivatives containing benzodioxole as potential anticancer agents. Bioorg Med Chem.

[56] Wang H, Zhang Y, Xie LP, Yu XY, Zhang RQ (2002). Effects of genistein and daidzein on the cell growth, cell cycle, and differentiation of human and murine melanoma cells. J Nutr Biochem.

[57] Wong RS (2011). Apoptosis in cancer: from pathogenesis to treatment. J Exp Clin Cancer Res.

[58] Zhang YH, Park YS, Kim TJ, Fang LH, Ahn HY, Hong JT, Kim Y, Lee CK, Yun YP (2000). Endothelium-dependent vasorelaxant and antiproliferative effects of apigenin. Vasc Pharmacol.

